# Influence of glycaemic control on macular thickness in diabetic retinopathy

**DOI:** 10.1002/edm2.308

**Published:** 2021-10-20

**Authors:** Sadhana Sharma, Pratap Karki, Sagun Narayan Joshi, Sanket Parajuli

**Affiliations:** ^1^ Mechi Eye Hospital Jhapa Nepal; ^2^ Department of Retina B.P Koirala Lions Center for Ophthalmic Studies (BPKLCOS) Institute of Medicine Kathmandu Nepal; ^3^ Reiyukai Eiko Masunaga Eye Hospital Banepa Nepal

**Keywords:** central macular thickness, diabetic macular oedema, HbA1c

## Abstract

**Introduction:**

Diabetic macular oedema (DME) is one of the major cause of decreased visual acuity in patients with diabetic retinopathy. Poor glycaemic control is associated with increased incidence of DME.

**Methods:**

A total of 112 eyes of 112 patients were studied in this cross‐sectional study and were classified into three groups based on HbA1c: group 1 included patients with good glycaemic control (HbA1c ≤7%), group 2 included patients with moderate glycaemic control (HbA1c between 7% and 9%) and group 3 included patients with poor glycaemic control (HbA1c ≥9%).

**Results:**

We included 112 eyes of 112 patients. The mean duration of diabetes mellitus (DM) was 11.37 years. In statistical analysis, CMT (mean 188.80 ± 27.64 μm) positively correlated with mean HbA1c level (7.95 ± 1.29%) (*r* = 0.238, *p *< .05). There was a significant difference in CMT values among the three groups of HbA1c (F (2,109) = 19.39, *p *< .001). Post hoc analysis showed statistical significance between HbA1c≤7% and HbA1c ≥9% group and HbA1c 7%–9% and ≥9% group. However, statistical significance was not found among HbA1c ≤7% group and HbA1c 7%–9% group. Multiple regression analyses showed a significant correlation between CMT and HbA1c after adjusting for age and duration of diabetes.

**Conclusion:**

Serum HbA1c level has a significant correlation with CMT in diabetic patients.

## Graphical abstract

Central macular thickness in diabetic retinopathy is correlated to glycosylated haemoglobin. A good glycaemic control can decrease macular oedema and other related complications.

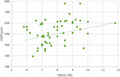

## 1 INTRODUCTION

Diabetic macular oedema (DME) is one of the major cause of poor visual acuity in diabetic retinopathy (DR).^1^ DME is characterized by increased vascular permeability and deposition of hard exudates at the central retina and can occur at any stage of DR. With the help of OCT, it is possible to quantify the macular thickness objectively and to follow the progression of DME quantitatively.^2^

Currently, DME is classified as ‘center involved’ or ‘non center involved’ (referring to whether or not there is oedema i.e., CMT>250 μm at the fovea) based on OCT. Non‐centre involved macular oedema is rarely symptomatic. However, as the oedema spreads to the central macula, patients usually experience progressive vision loss and treatment with anti‐VEGF is recommended.^3^, ^4^, ^5^

Glycated haemoglobin (HbA1c) measurements reflect the long‐term control of hyperglycaemia. Higher levels of HbA1c increased the incidence of DME over a 10‐year period in Wisconsin Epidemiology Study of Diabetic Retinopathy (WESDR).^6^ The Diabetes Control and Complication Trial (DCCT) and the UK Prospective Diabetic Study (UKPDS) both of which were randomized prospective studies showed that intensive glycaemic control and thus reduction of HbA1c levels are associated with a decrease in the rates of development and progression of DR and DME.^7^

The purpose of this study is to evaluate the relationship between HbA1c and CMT measured by OCT in patients with diabetic retinopathy.

## 2 METHODS

Patients with Type 2 DM with centre‐involving DME (Oedema involving the fovea and CMT > 250 μm)^4^ were included in the study between January 2017 and June 2018. Approval was taken from Institutional review committee of Institute of Medicine. All research procedures followed the tenets of the Declaration of Helsinki. Informed consent was obtained from every enrolled subject.

Exclusion criteria were as follows: 1. Significant cataract or other media opacities that interfere with fundus evaluation 2. Retinal pathologies such as retinal vascular occlusions, Age‐related macular degeneration 3. History of photocoagulation 4. History of prior intravitreal anti‐VEGF injections.

Demographic details were taken, and ocular examination was done which included best corrected visual acuity (BCVA) taken by Snellen chart and converted to logarithm of minimum angle of resolution (log MAR), anterior segment evaluation under slit lamp, and intraocular pressure measurement with the Goldman applanation tonometer. Detailed fundoscopic evaluation was done after pupillary dilatation with 0.8% tropicamide and 5% phenylephrine eye drops using indirect ophthalmoscopy with +20D lens and slit lamp bio microscopy with +90D lens. The grading of diabetic retinopathy was done as per the ‘International clinical diabetic retinopathy disease severity scale’.^8^

Baseline CMT was measured using Spectral Domain OCT (SD‐OCT) (OCT Spectralis Heidelberg engineering). All OCT scans were performed by the same experienced operator. Macular thickness was taken in both eyes but the eye with higher macular thickness was used for statistical analysis.

Baseline Serum HbA1c was measured using ion exchange resin method. Patients were stratified into three groups based on HbA1c values—Good control (HbA1c ≤7%), Moderate control (HbA1c 7%–9%) and Poor control (HbA1c ≥9%). These categorical values were based on the DCCT trial.

### 2.1 Data processing and statistical analysis

Continuous data were expressed in terms of mean ± Standard Deviation (SD). Kolmogorov‐Smirnov test was used to check normality of the data. A correlation test (Pearson coefficient when the variables had normal distribution or Spearman coefficient when the distribution of variables was not normal) was used to evaluate the correlation between CMT and HbA1c, Duration of diabetes, log MAR visual acuity, etc. Comparison of macular thickness among three groups defined by HbA1c was done using one‐way analysis of variance (ANOVA) test. For post hoc analysis, Tukey test was used. Multiple regression analysis (enter method) was used to analyse the relationship between a dependent variable and one or more independent variables. Probability values of *p* < .05 were considered statistically significant.

All statistical calculations were done using computer programs Microsoft Excel 2016 and SPSS V.23 for Microsoft Windows.

## 3 RESULTS

One hundred twelve eyes of 112 patients with DME were included in this cross‐sectional study. Sixty‐four patients were male and 38 were female. The mean age ± SD was 55.56 ± 9.22 years (range 36–80 years). The mean DM duration was 11.37 ± 5.32 years (range, 1–25 years). The mean value of HbA1c was 7.69 ± 1.12% (range, 5.8%–11.8%). Right eye was affected more than left eye in 67 (59.82%) cases.

Thirty‐eight (33.9%) of patients had moderate non‐proliferative diabetic retinopathy (NPDR), 63(56.3%) had severe NPDR and 11(9.8%) had proliferative diabetic retinopathy (PDR) as shown in Table 1.

**TABLE 1:** Baseline characteristics of the study population

Parameters	Values
Age (Mean ± SD) (years)	55.56 ± 9.22
Sex (Male/Female)	64/38
Laterality (Right/Left)	67/45
Duration of Diabetes (Mean ± SD) (years)	11.37 ± 5.32
HbA1c (Mean ± SD) (%)	7.69 ± 1.12
CMT (Mean ± SD) (μm)	493.54 ± 101.94
Grade of Diabetic retinopathy (Moderate NPDR/Severe NPDR/PDR)	38/63/11

Abbreviations: CMT, central macular thickness; HbA1c, glycated haemoglobin; NPDR, non‐proliferative diabetic retinopathy; PDR, proliferative diabetic retinopathy; SD, standard deviation.

Baseline characteristics of the three study groups are shown in Table 2. The mean CMT and log MAR VA in three groups (group 1, group 2 and group 3) were 476.36 ± 102.53, 443.50 ± 69.02 and 566.33 ± 92.82 microns and 0.83 ± 0.45, 0.75 ± 0.23, 0.95 ± 0.36 log units, respectively.

**TABLE 2:** Baseline characteristics of the study groups

	HbA1c ≤7% (*n* = 36)	HbA1c 7%–9% (*n* = 40)	HbA1c ≥9% (*n* = 36)	*p* value
Age (years)	54.30 ± 10.01	58.13 ± 9.06	53.97 ± 7.97	.084
CMT (μm)	476.36 ± 102.53	443.50 ± 69.02	566.33 ± 92.28	<.001
Log MAR VA (log units)	0.83 ± 0.45	0.75 ± 0.23	0.95 ± 0.36	.063
Duration of DM (years)	9.56 ± 2.54	12.68 ± 6.78	11.72 ± 3.88	.033

Abbreviations: CMT, central macular thickness; DM, diabetes mellitusHbA1c, glycated haemoglobin; Log MAR, logarithm of minimum angle of resolution; VA, visual acuity.

**FIGURE 1:**
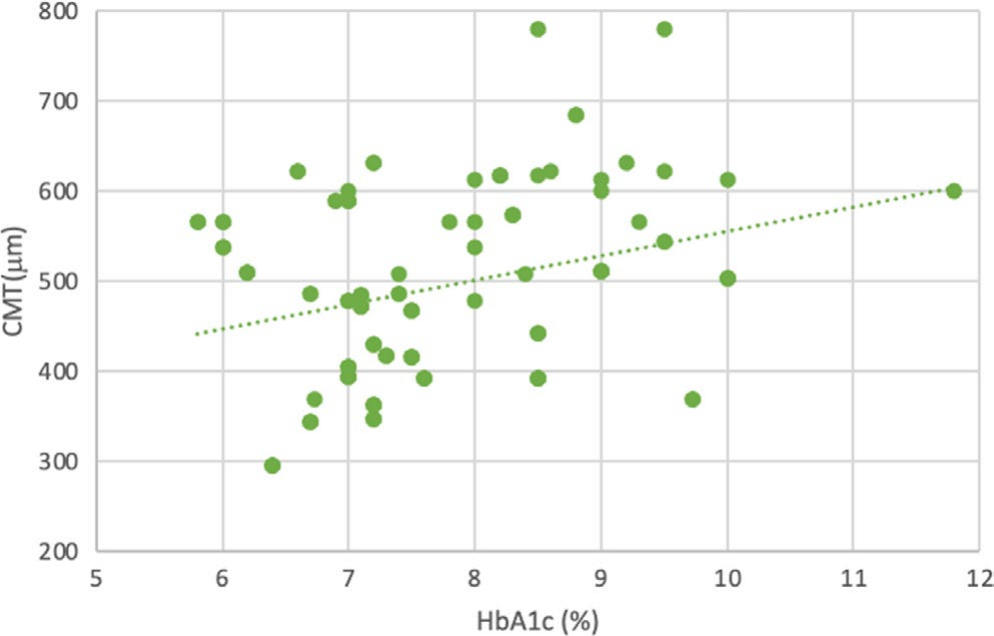
Scatter diagram showing correlation between HbA1c and central macular thickness

Mean baseline CMT was 493.54 ± 101.94 microns (range, 296–780). A positive correlation was found between HbA1c and CMT (*r* = 0.238, *p *< .05) (Figure 1).

Also, a positive correlation was seen between baseline CMT and baseline log MAR visual acuity. (*r* = 0.641, *p *< .001). There was no statistically significant relation between HbA1c and log MAR visual acuity through positive correlation was seen (*r* = 0.147, *p *> .05).

**FIGURE 2:**
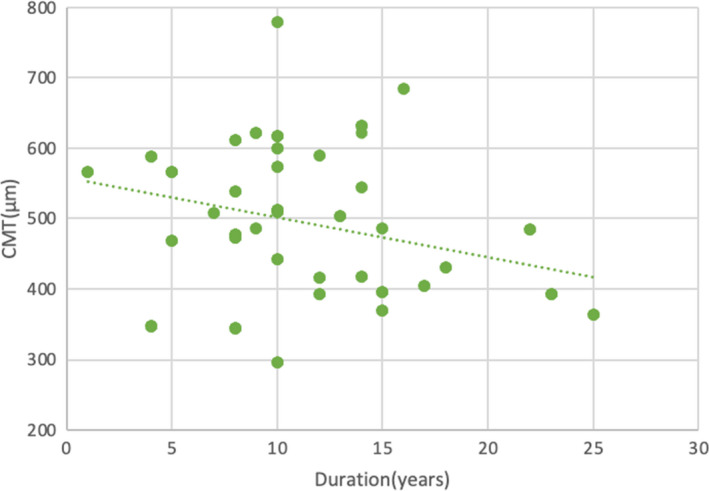
Scatter diagram showing correlation between duration of diabetes and central macular thickness

We also assessed the relation between duration of diabetes and CMT. A statistically significant negative correlation was found. (*r* = −0.256, *p *> .05) (Figure 2).

There were 36 patients with HbA1c ≤7%, 40 patients with HbA1c 7%–9% and 36 patients with HbA1c ≥9%. Baseline CMT was found to be statistically significant among three groups (F (2, 109) = 19.39, *p *< .001). On a post hoc test, statistical significance was found between HbA1c ≤7% and HbA1c ≥9% group (*p *< .001) and HbA1c 7%–9% and ≥9% group (*p *< .001). However, no statistical significance was found among HbA1c ≤7% and HbA1c 7%–9% group (*p *= .242) (Table 3).

**TABLE 3:** Post hoc test

HbA1c (%)	*p* value
Group 1 and Group 2	.242
Group 2 and Group 3	<.001
Group 1 and Group 3	<.001

Abbreviation: HbA1c, glycated haemoglobin.

Multiple regression analyses (enter method) were performed with central macular thickness as a dependent variable and with age, HbA1c and duration of diabetes as independent variable. A significant correlation persisted between central macular thickness and HbA1c and between central macular thickness and duration of diabetes (Table 4).

**TABLE 4:** Multiple regression analysis of central macular thickness as a dependent variable and HbA1c and duration of diabetes as independent variable

	*β* coefficient	Standard error	*t* value	*p* value
HbA1c (%)	0.34	7.98	3.92	<.001
Duration of DM (years)	−0.33	1.72	−3.76	<.001
Age (years)	0.12	1.09	1.39	.167

Abbreviations: DM, diabetes mellitusHbA1c, glycated haemoglobin.

## 4 DISCUSSION

American Diabetes Association recommends HbA1c goal for non‐pregnant adults of <7% (53 mmol/mol).^9^ Achieving HbA1c targets of <7% (53 mmol/mol) has been shown to reduce microvascular complications in the long term when started early in the course of disease.^10^ Good glycaemic control and blood pressure are considered the most important modifiable risk factors to reduce the risk of progression of DR and vision loss among the several associated risk factors.^11^

In our study, we assessed the relation between central macular thickness and glycated haemoglobin in patients with diabetic retinopathy. We found that HbA1c is positively correlated with macular thickness. On comparing CMT values between categories of HbA1c, least macular thickness was found in the group with good glycaemic control.

A study by Moreira et al^12^ found the only variable that showed a significant association with macular oedema was HbA1c. Also, a study by Chou et al showed HbA1c of 8 or over was associated with an increased risk of clinically significant macular oedema (CSME) in diabetic eyes and there was a significant correlation between younger age, shorter DM duration and higher macular thickness. However, the cut‐off value for HbA1c in this study was 8% and CSME was only diagnosed when central macular thickness was greater than 325 μm in OCT. A study by Baharak Asefzadeh et al did not show a significant correlation between HbA1c and macular thickness.^13^

In a retrospective study by Yi‐Jie Peng et al,^14^ patients with baseline HbA1c of 7% or less had significantly higher CMT compared with those with baseline HbA1c more than 7%.^14^

A significant negative correlation was seen between duration of diabetes and CMT, and this was similar to the findings in the study by Baharak Asefzadeh et al.^13^ But only diabetic patients with no retinopathy and mild retinopathy were evaluated in this study.

Microvascular dysfunction is the most commonly accepted pathophysiological model for development of diabetic retinopathy. In addition to microvascular changes, diabetic retinopathy seems to involve neurodegenerative changes as shown by electroretinographic and psychophysical studies and these have demonstrated that retinal function is altered in diabetic patients and is related to duration of the disease.^13^

It is well known that longer duration of diabetes is a risk factor for the development of diabetic retinopathy and DME. Nevertheless, a decreased macular thickness with longer duration of diabetes as seen in our study may reflect neurodegenerative processes in the retina and could be the result of ganglion cell or glial cell structural alterations.

In conclusion, there was a statistically significant correlation between glycaemic control and macular thickness. Early screening and good glycaemic control from the time of diagnosis can decrease the risk of diabetic macular oedema and related complications.

## CONFLICT OF INTEREST

The authors declare that they have no competing interests.

## AUTHOR CONTRIBUTIONS

**Pratap Karki:** Conceptualization (equal); Investigation (equal); Methodology (equal); Supervision (equal); Validation (equal); Writing‐review & editing (supporting). **Sagun Joshi:** Conceptualization (equal); Methodology (equal); Supervision (equal); Writing‐review & editing (supporting). **Sanket Parajuli:** Conceptualization (equal); Formal analysis (equal); Methodology (equal); Resources (equal); Software (equal); Supervision (equal); Writing‐review & editing (equal).

## ETHICAL APPROVAL AND CONSENT TO PARTICIPATE

The study was performed in accordance with the tenets of the Declaration of Helsinki after obtaining necessary consent from participants before the study. Ethics approval was obtained from Institutional Review Committee (IRC) of Institute of Medicine (IOM), Tribhuvan University (TU).

## Data Availability

Data sets generated during and/or analysed during the current study are available from the corresponding author on request.
